# Constitutional variants in *PTEN*: a frequent finding in patients with papillary tumors of the pineal region subtype B (PTPR-B) associated with isolated loss of chromosome 10

**DOI:** 10.1007/s00401-025-02865-8

**Published:** 2025-03-14

**Authors:** Steffen Hirsch, Ramin Rahmanzade, Kerstin Grund, Christian Sutter, Kathrin Schramm, Florian Selt, Jonas Ecker, Barbara C. Jones, Daniel Schrimpf, Martin Demmert, Ana S. Guerreiro Stücklin, Pablo Hernaiz Driever, Markus Mezger, Ines Brecht, Sasan D. Adib, Bastian Brummel, Dominik Sturm, Nicola Dikow, Maja Hempel, Till Milde, Kristian Pajtler, David T. W. Jones, Stefan M. Pfister, Andreas von Deimling, Felix Sahm, Christian P. Schaaf

**Affiliations:** 1 Institute of Human Genetics, Heidelberg University, Heidelberg, Germany; 2 Hopp Children’s Cancer Center Heidelberg (KiTZ) and Division of Pediatric Neurooncology, German Cancer Research Center (DKFZ), German Consortium for Translational Cancer Research (DKTK), Heidelberg, Germany; 3 Department of Neuropathology, Institute of Pathology, University Hospital Heidelberg, Heidelberg, Germany; 4 Clinical Cooperation Unit Neuropathology, German Cancer Research Center (DKFZ), German Consortium for Translational Cancer Research (DKTK), Heidelberg, Germany; 5 Department of Pediatric Oncology, Hematology, Immunology and Pulmonology, University Hospital Heidelberg, Heidelberg, Germany; 6 Hopp Children’s Cancer Center Heidelberg (KiTZ) and Division of Pediatric Glioma Research, German Cancer Research Center (DKFZ), Heidelberg, Germany; 7 Hopp Children’s Cancer Center Heidelberg (KiTZ) and Clinical Cooperation Unit Pediatric Oncology, German Cancer Research Center (DKFZ), German Consortium for Translational Cancer Research (DKTK), Heidelberg, Germany; 8 Department of Pediatric Oncology, University Hospital Schleswig Holstein - Lübeck, Lübeck, Germany; 9 Department of Oncology and Children’s Research Center, University Children’s Hospital Zurich, Zurich, Switzerland; 10 Department of Pediatric Oncology and Hematology, Charité-Universitätsmedizin Berlin, Berlin, Germany; 11 Department of General Pediatrics, Hematology and Oncology, University Children’s Hospital, Tübingen, Germany; 12 Department of Neurosurgery, University of Tuebingen, Tübingen, Germany; 13 Department of Pediatric Oncology and Hematology, Klinikum Dortmund, Dortmund, Germany; 14 Department of Pediatrics, Jena University Hospital and Comprehensive Cancer Center Central Germany (CCCG), Jena, Germany

Papillary tumors of the pineal region (PTPR) have been recognized as a distinct tumor type with characteristic histopathological features and a distinct DNA methylation profile [8, 9]. Recent studies have further elucidated the molecular and cytogenetic etiologies of PTPRs, revealing common chromosomal and genetic alterations that may contribute to our understanding of their origins and potential treatment strategies [1, 2, 5]. PTPRs can be divided into two distinct methylation subclasses, known as PTPR-A and PTPR-B. Tumors of both subclasses very frequently show loss of chromosome 10, with up to 25% of PTPR-B showing additional somatic alterations of *PTEN* [5–11]. The PTPR-B subgroup has recently been further classified into two distinct epigenetic subclasses, PTPR-B1 and PTPR-B2 with distinct copy number alterations and clinical courses. Tumors in the PTPR-B1 subclass frequently demonstrate loss of chromosomes 3 and 14 and are associated with a more favorable prognosis compared to those in the PTPR-B2 subclass [16].

We recently published a patient with PTPR and a constitutional variant in the *PTEN* gene [13]. However, to our knowledge, until now there was no information regarding the prevalence of constitutional *PTEN* variants among patients with PTPR-B.

To address this, we identified individuals with PTPR-B in four pediatric and young adult cancer patient registry cohorts: MNP2.0 (*n* = 1034), MNP Int-R (*n* = 806), PTT (*n* = 582), INFORM (*n* = 2909). All patients included in this analysis had been investigated by a multi-omics approach consisting of tumor DNA-methylation analysis (Illumina Infinium DNA methylation array) and paired tumor/blood sequencing using multi-gene panel (MNP2.0, MNP Int-R, PTT) or whole exome/whole genome sequencing (INFORM). Tumor copy number variation (CNV) profiles were generated using DNA methylation data. Germline sequencing data were analyzed and classified using virtual panels of established cancer predisposition genes, as previously reported [4, 13, 14]. All registries were approved by the local ethics committee.

We identified ten patients with PTPR-B in our cohorts (*f*/*m*: 6/4; age at diagnosis: 0–24 years, mean: 8 years). The demographic, clinical and molecular findings of patients are summarized in Table 1. Germline sequencing showed a constitutional heterozygous pathogenic or likely pathogenic (P/LP) *PTEN* variant in five individuals. Variants were either truncating (*n* = 4) or missense within the phosphatase domain (*n* = 1). Those with germline variants were significantly younger at diagnosis than those without germline variants (mean age at diagnosis 2.2 vs 14.2 years, *p* = 0.014). All patients with *PTEN* germline variant were under 5 years of age, while all patients without *PTEN* germline variant were 6 years or older. All patients with germline variants and available clinical information showed clinical signs of PTEN hamartoma tumor syndrome (PHTS; Table 1). In one patient (#6), PHTS was already known at the time of PTPR diagnosis.

**Table 1:** The demographic, clinical and diagnostic findings of patients with PTPR-B

Identifier		Clinical information			Somatic alterations			Sequencing			
#	Registry	Sex	Age at diagnosis (years)	Clinical signs of PHTS	DNA methylation class	CNV at diagnosis	CNV at relapse	Variant (NM_000314.4)	VAF Control	VAF Tumor	ACMG class
1	PTT2.0	f	3.2	Macrocephaly, thyroid disease, trichilemmoma, fibroma	PTPR-B2	n/a	Isolated loss of Chr. 10	c.1023delT p.(Phe341Leufs*3)	0.35	0.78	pathogenic
2	PTT2.0	m	24.0	n/a	PTPR-B1	Loss of Chr. 3, 10, 14q, 22q; gain of Chr. 8, 16, 20	n/a	No variant detected	n/a	n/a	n/a
3	MNP2.0	f	14.0	n/a	PTPR-B1	Loss of Chr. 10; gain of Chr. 8, 9, 12, 19	n/a	c.634 + 1G > C p.?	0	0.5	n/a
4	MNP2.0*	m	0.6	unknown	PTPR-B2	Isolated loss of Chr. 10	n/a	c.80-1_80del p.?	0.5	0.58	likely pathogenic
5	MNP2.0	f	16.0	n/a	PTPR-B2	Loss of Chr. 10; gain of Chr. 8	n/a	No variant detected	n/a	n/a	n/a
6	INFORM	m	4.0	Macrocephaly, thyroid disease, haemangioma, fibroma, developmental delay, autism spectrum disorder	PTPR-B1	Loss of Chr. 3, 10	Isolated loss of Chr. 10	c.604del p.(Thr202Leufs*19)	0.56	0.73	likely pathogenic
7	INFORM	f	11.0	n/a	PTPR-B2	n/a	Loss of Chr. 3, 10, 18; gain of Chr. 8, 12	No variant detected	n/a	n/a	n/a
8	INFORM	f	6.0	n/a	PTPR-B2	n/a	Loss of Chr. 10; gain of Chr. 4, 8, 9, 11,12	c.394G > T p.(Gly132Cys)	0	0.74	n/a
9	MNP Int-R	f	0.6	Macrocephaly	PTPR-B2	n/a	Isolated loss of Chr. 10	c.464A > G p.(Tyr155Cys)	0.44	0.82	pathogenic
10	INFORM	m	2.6	Macrocephaly, developmental delay	PTPR-B2	Isolated loss of Chr. 10	Loss of Chr. 10; gain of Chr. 8, 13, 17	c.634 + 5G > C p.?	0.5	0.84	pathogenic

Abbreviations: *ACMG* American College of Medical Genetics, *n/a* not available, *PHTS* PTEN hamartoma tumor syndrome, *CNV* copy number variation, *VAF* variant allele fraction

^*^Case previously published [13]

We compared DNA methylation data from our cohort with a reference set of 172 PTPR tumors that were confidently classified as PTPR-A or PTPR-B (match score > 0.90) based on a previously published classification algorithm [2] from the internal database of the Neuropathology Department, Heidelberg University. Using a t-SNE analysis of the combined cohort we validated findings from a recent study that identified two epigenetic subclasses (PTPR-B1 and PTPR-B2) within PTPR-B at a larger scale (Supplementary Figs. 1 and 2) [16]. Notably, four out of five patients with constitutional P/LP *PTEN* variants clustered within the PTPR-B2 subclass.

Copy number analysis of tumor samples showed loss of chromosome ten including *PTEN* in all cases and additional copy number changes (e.g. loss of chromosome 3, gain of chromosome 8) in six cases. Loss of chromosome 10 was the only chromosomal alteration in the CNV profile of 4 out of 5 individuals with a constitutional P/LP *PTEN* variant, while tumors from patients lacking constitutional *PTEN* alterations exhibited a broader spectrum of chromosomal alterations. Figure 1 provides an overview of the radiological, histopathological, and molecular findings for patient # 9 (Fig. 1a–d) and additionally compares the cumulative CNV profiles of the entire cohort (*n* = 10) with those of the patients with constitutional P/LP *PTEN* variants (Fig. 1e). Only one patient with constitutional P/LP *PTEN* variant (#6) was identified to show additional CNVs (loss of chromosome 10 and 3) in the tumor analysis at diagnosis, interestingly this is the same patient mapping to the PTPR-B1 subclass and the oldest patient in our cohort with a constitutional variant. At recurrence the tumor exhibited an isolated loss of chromosome 10.

**Fig. 1:**
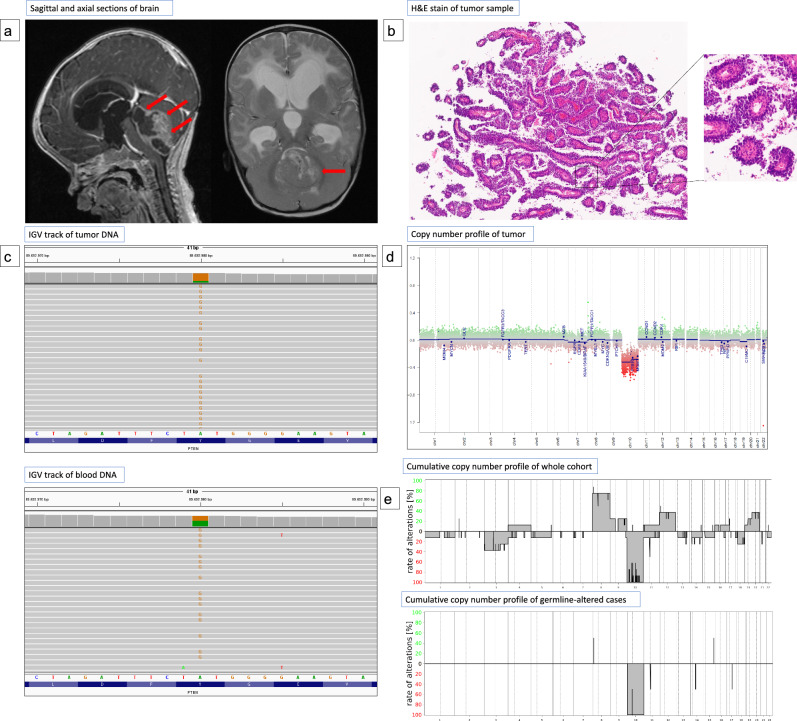
**a** Sagittal contrast-enhanced and axial T2-weighted magnetic resonance (MR) sections of a 7-month-old child (# 9) depicting a large pineal mass and hydrocephalus **b** Hematoxylin and eosin (H&E) staining reveals characteristic papillary architecture, frequent mitoses, and necrosis (not shown), suggesting Papillary Tumor of the Pineal Region (PTPR). **c** Next-generation sequencing (NGS) of control DNA (bottom panel) identified a heterozygous constitutional pathogenic *PTEN* variant showing loss of heterozygosity in tumor DNA (top panel). **d** DNA methylation analysis showed isolated loss of chromosome 10 in copy number profile. **e** Histogram illustrating the frequency of chromosomal alterations in the overall cohort (*n* = 10, top) compared to those with constitutional *PTEN* variants (*n* = 5, bottom)

Our findings reveal the frequent occurrence of constitutional P/LP *PTEN* variants in pediatric PTPR-B, underscoring the gene’s pivotal role in tumor development. Additionally, isolated loss of chromosome 10 appears to be intricately linked with these germline alterations, suggesting that loss of PTEN function may be sufficient for tumorigenesis in this subset. These genetic insights may also have profound implications for therapeutic strategies, Targeting the PI3K/AKT/mTOR pathway may be possible in these patients. At present there is not enough data regarding the safety and efficacy of mTOR pathway inhibition in patients with PHTS and further pre-clinical and clinical evidence is required [3].

This work expands the spectrum of CNS tumors associated with PHTS that had previously been limited to benign tumors like Lhermitte Duclos disease. We suspect that PHTS is underdiagnosed in patients with PTPR as many signs or symptoms of PHTS may be falsely attributed to tumor or treatment effects. For example, macrocephaly may exclusively be attributed to hydrocephalus at tumor presentation, developmental delay may be considered to be secondary to the space-occupying lesion and thyroid disease may be considered an adverse effect of radiotherapy.

Currently, no data are available on the prevalence of PTPR among patients with PTEN hamartoma tumor syndrome. Even within the spectrum of PHTS, PTPRs are very likely a rare event. A recent review of the literature identified only 25 patients with intracranial tumors, including one case with an unspecified tumor of the pineal region [10, 12]. Imaging studies should be considered with a low threshold if symptoms consistent with an intracranial space-occupying lesion are present in young patients with PHTS.

Our findings warrant validation in larger cohorts, especially as the availability of clinical and follow-up information is limited in our registries. Larger cohorts may also allow to identify a subgroup of PHTS patients at an increased risk of developing PTPR.

As discussed above signs and symptoms of PHTS may not be recognized as such or may not yet be present at time of tumor diagnosis. Early detection of a tumor predisposing syndrome like PHTS is important, given the implications for the further management of the patient and potentially other family members [15, 17]. We propose that genetic counseling and potentially evaluation for constitutional *PTEN* variants should be considered in all patients with PTPR-B2 with isolated loss of chromosome 10 or a young age at diagnosis.

## Supplementary Information

Below is the link to the electronic supplementary material.

**Supplementary Fig. 1:** Unsupervised, non-linear t-distributed stochastic neighbor embedding (t-SNE) projection of DNA methylation array profiles from 182 PTPR tumor samples including cohort tumors (*n* = 10), a reference set of PTPR-A tumors (*n* = 52), and PTPR-B tumors (*n* = 120). The analysis recapitulates the recently proposed epigenetic subclasses of PTPR-B (PTPR-B1 and -B2) with distinct copy number profiles at a larger scale. Furthermore, 4 out of 5 patients with constitutional *PTEN* variants clustered within PTPR-B2. (PNG 117 KB)

**Supplementary Fig. 2:** Cumulative summary of copy number variations (CNVs) across methylation subgroups highlights distinct CNV profiles for PTPR-B1 and PTPR-B2. Consistent with previous findings, tumors in the PTPR-B1 subgroup frequently exhibit chromosome losses in chromosomes 3 and 14. (PDF 179 KB)

## References

[1] Assi HI, Kakati RT, Berro J, Saikali I, Youssef B, Hourany R et al (2021) PTEN R130Q papillary tumor of the pineal region (PTPR) with chromosome 10 loss successfully treated with everolimus: a case report. Curr Oncol 28(2):1274–1279. 10.3390/curroncol28020121 33804593 10.3390/curroncol28020121 PMC8025816

[2] Capper D, Jones DTW, Sill M, Hovestadt V, Schrimpf D, Sturm D et al (2018) DNA methylation-based classification of central nervous system tumours. Nature 555(7697):469–474. 10.1038/nature26000 29539639 10.1038/nature26000 PMC6093218

[3] Dhawan A, Baitamouni S, Liu D, Yehia L, Anthony K, McCarther A et al (2025) Cancer and overgrowth manifestations of PTEN hamartoma tumour syndrome: management recommendations from the international PHTS consensus guidelines working group. Clin Cancer Res. 10.1158/1078-0432.CCR-24-3819 39937242 10.1158/1078-0432.CCR-24-3819 PMC12010961

[4] Ecker J, Selt F, Sturm D, Sill M, Korshunov A, Hirsch S et al (2023) Molecular diagnostics enables detection of actionable targets: the pediatric targeted therapy 2.0 registry. Eur J Cancer (Oxford, England: 1990) 180:71–84. 10.1016/j.ejca.2022.11.015 10.1016/j.ejca.2022.11.015 36542877

[5] Goschzik T, Gessi M, Denkhaus D, Pietsch T (2014) PTEN mutations and activation of the PI3K/Akt/mTOR signaling pathway in papillary tumors of the pineal region. J Neuropathol Exp Neurol 73(8):747–751. 10.1097/NEN.0000000000000093 25003235 10.1097/NEN.0000000000000093

[6] Gutenberg A, Brandis A, Hong B, Gunawan B, Enders C, Schaefer IM et al (2011) Common molecular cytogenetic pathway in papillary tumors of the pineal region (PTPR). Brain Pathol 21(6):672–677. 10.1111/j.1750-3639.2011.00493.x 21470326 10.1111/j.1750-3639.2011.00493.x PMC8094051

[7] Hasselblatt M, Blümcke I, Jeibmann A, Rickert CH, Jouvet A, van de Nes JA et al (2006) Immunohistochemical profile and chromosomal imbalances in papillary tumours of the pineal region. Neuropathol Appl Neurobiol 32(3):278–283. 10.1111/j.1365-2990.2006.00723.x 16640646 10.1111/j.1365-2990.2006.00723.x

[8] Heim S, Sill M, Jones DT, Vasiljevic A, Jouvet A, Fèvre-Montange M et al (2016) Papillary tumor of the pineal region: a distinct molecular entity. Brain Pathol 26(2):199–205. 10.1111/bpa.12282 26113311 10.1111/bpa.12282 PMC8029206

[9] Louis DN, Perry A, Wesseling P, Brat DJ, Cree IA, Figarella-Branger D et al (2021) The 2021 WHO classification of tumors of the central nervous system: a summary. Neuro Oncol 23:1231–1251. 10.1093/neuonc/noab106 34185076 10.1093/neuonc/noab106 PMC8328013

[10] Lynch ED, Ostermeyer EA, Lee MK, Arena JF, Ji H, Dann J et al (1997) Inherited mutations in PTEN that are associated with breast cancer, cowden disease, and juvenile polyposis. Am J Hum Genet 61(6):1254–1260. 10.1086/301639 9399897 10.1086/301639 PMC1716102

[11] Pfaff E, Aichmüller C, Sill M, Stichel D, Snuderl M, Karajannis MA et al (2020) Molecular subgrouping of primary pineal parenchymal tumors reveals distinct subtypes correlated with clinical parameters and genetic alterations. Acta Neuropathol 139(2):243–257. 10.1007/s00401-019-02101-0 31768671 10.1007/s00401-019-02101-0 PMC7275775

[12] Prieto R, Hofecker V, Corbacho C (2023) Coexisting lipomatous meningioma and glioblastoma in Cowden syndrome: a unique tumor association. Neuropathology 43(1):110–116. 10.1111/neup.12858 36003032 10.1111/neup.12858

[13] Sturm D, Capper D, Andreiuolo F, Gessi M, Kölsche C, Reinhardt A et al (2023) Multiomic neuropathology improves diagnostic accuracy in pediatric neuro-oncology. Nat Med 29(4):917–926 36928815 10.1038/s41591-023-02255-1 PMC10115638

[14] van Tilburg CM, Pfaff E, Pajtler KW, Langenberg KPS, Fiesel P, Jones BC et al (2021) The pediatric precision oncology INFORM registry: clinical outcome and benefit for patients with very high-evidence targets. Cancer Discov 11(11):2764–2779 34373263 10.1158/2159-8290.CD-21-0094 PMC9414287

[15] Tischkowitz M, Colas C, Pouwels S, Hoogerbrugge N, PHTS Guideline Development Group, & European Reference Network GENTURIS (2020) Cancer surveillance guideline for individuals with PTEN hamartoma tumour syndrome. Eur J Hum Genetics 28(10):1387–1393. 10.1038/s41431-020-0651-7 32533092 10.1038/s41431-020-0651-7 PMC7608293

[16] Wu Z, Dazelle K, Abdullaev Z, Chung HJ, Dahiya S, Wood M et al (2024) Papillary tumor of the pineal region: analysis of DNA methylation profiles and clinical outcomes in 76 cases. Acta Neuropathol Commun 12(1):117. 10.1186/s40478-024-01781-4 39014393 10.1186/s40478-024-01781-4 PMC11251120

[17] Yehia L, Eng C (2001) PTEN Hamartoma Tumor Syndrome. In: Adam MP et al (eds) GeneReviews^®^. University of Washington, Seattle 20301661

