# Supplementary figures and images for: Constitutional variants in PTEN: a frequent finding in patients with papillary tumors of the pineal region subtype B (PTPR-B) associated with isolated loss of chromosome 10
Source: Acta Neuropathol. 2025 Mar 14;149(1):25. doi: 10.1007/s00401-025-02865-8 (PMC11909084; doi:10.1007/s00401-025-02865-8)

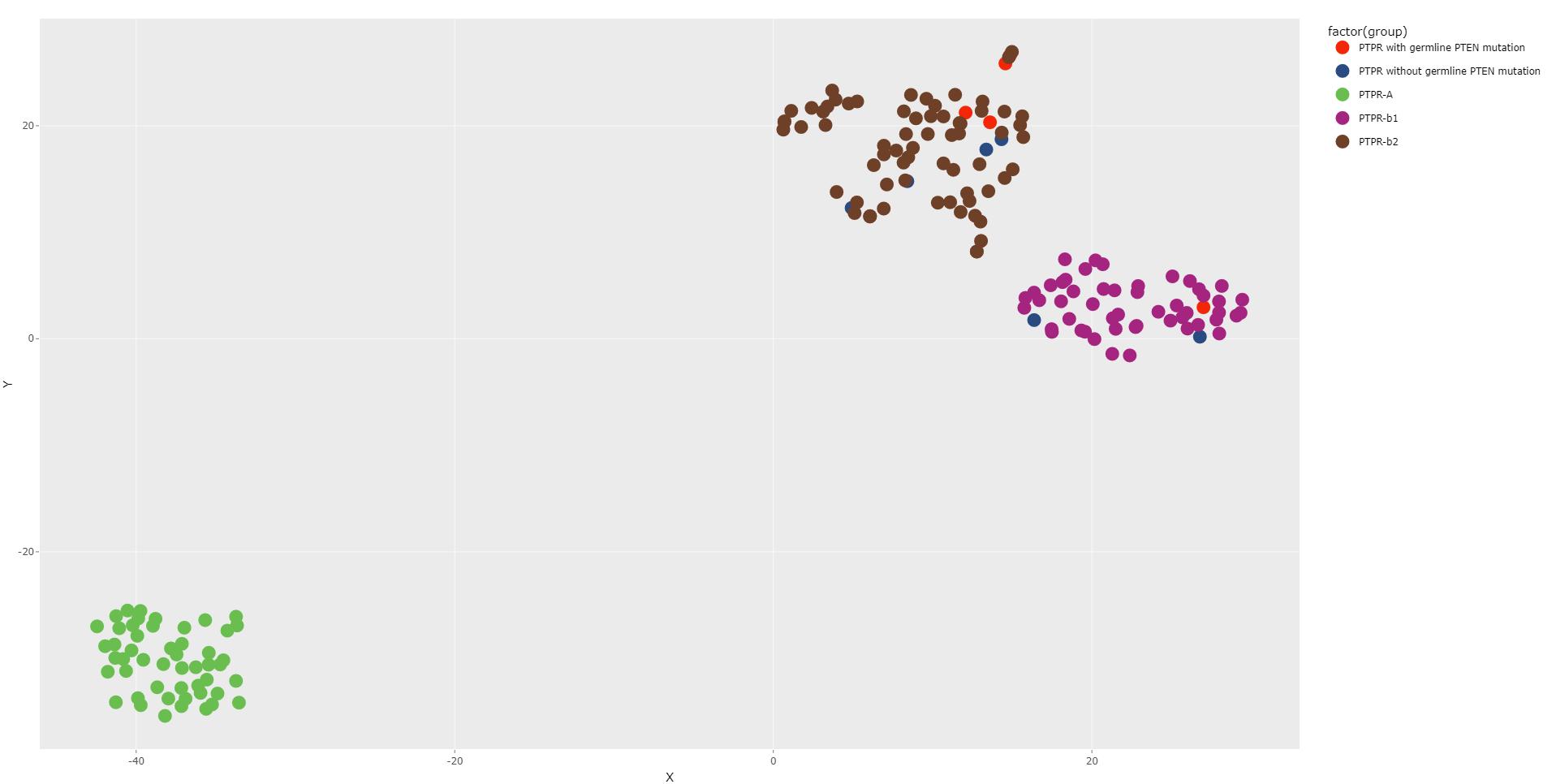

Supplement: Supplementary file 1 — Supplementary Fig. 1: Unsupervised, non-linear t-distributed stochastic neighbor embedding (t-SNE) projection of DNA methylation array profiles from 182 PTPR tumor samples including cohort tumors (n = 10), a reference set of PTPR-A tumors (n = 52), and PTPR-B tumors (n = 120). The analysis recapitulates the recently proposed epigenetic subclasses of PTPR-B (PTPR-B1 and -B2) with distinct copy number profiles at a larger scale. Furthermore, 4 out of 5 patients with constitutional PTEN variants clustered within PTPR-B2. (PNG 117 KB) [file 401_2025_2865_MOESM1_ESM.png]

a

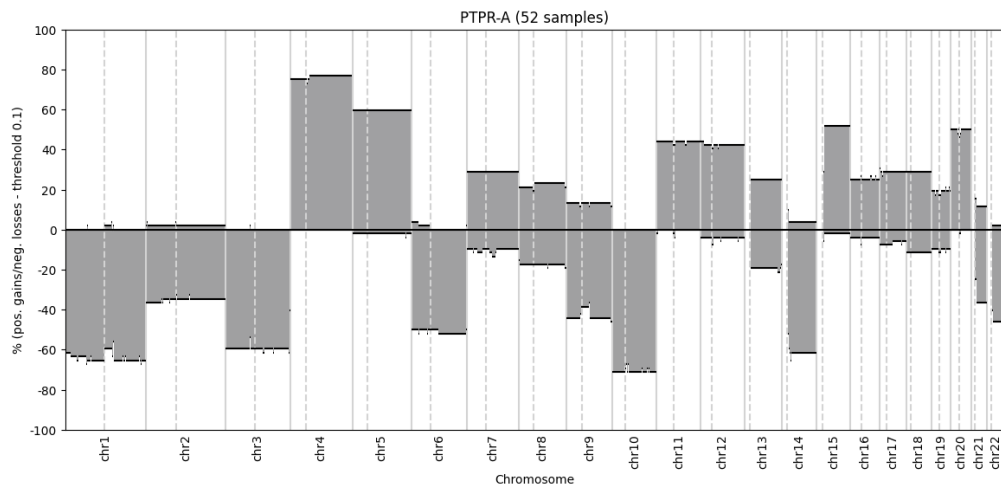

b

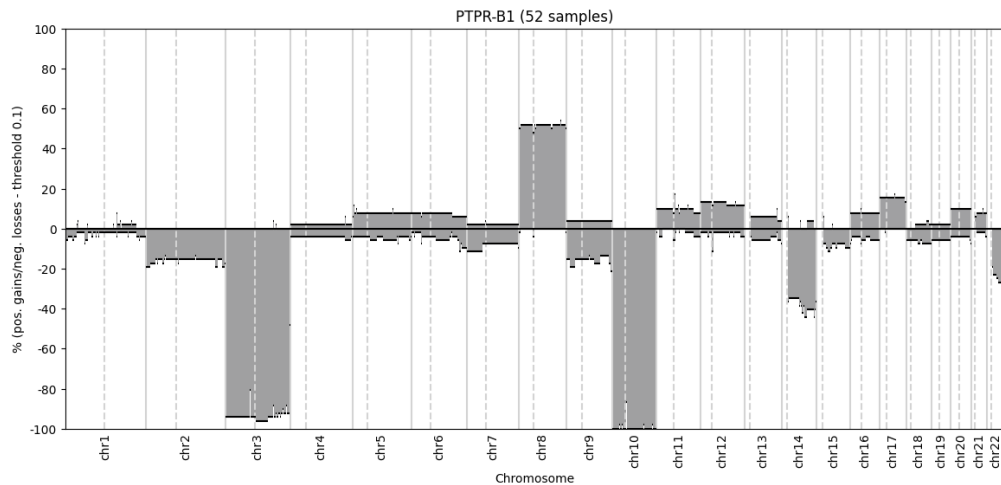

c

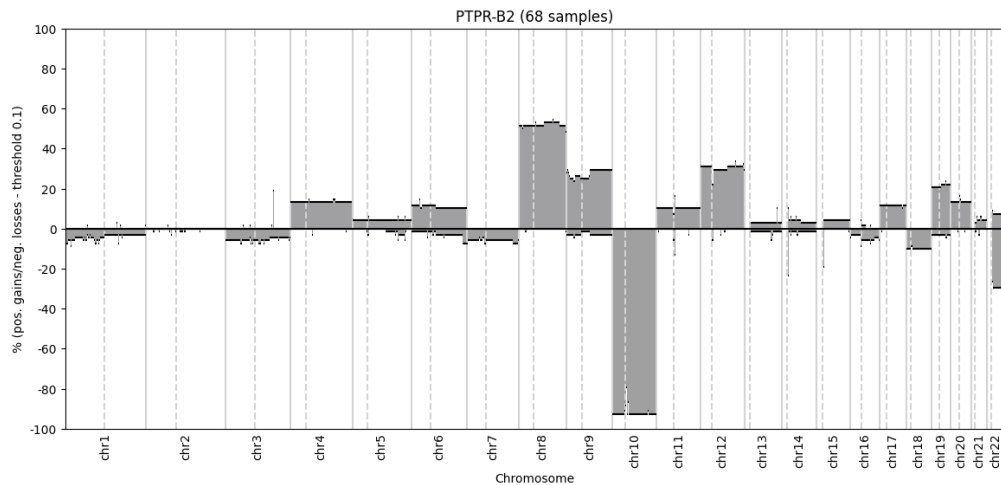

Supplement: Supplementary file 2 — Supplementary Fig. 2: Cumulative summary of copy number variations (CNVs) across methylation subgroups highlights distinct CNV profiles for PTPR-B1 and PTPR-B2. Consistent with previous findings, tumors in the PTPR-B1 subgroup frequently exhibit chromosome losses in chromosomes 3 and 14. (PDF 179 KB) [file 401_2025_2865_MOESM2_ESM.pdf]
